# Immunolocalization of Anti-Hsf1 to the Acetabular Glands of Infectious Schistosomes Suggests a Non-Transcriptional Function for This Transcriptional Activator

**DOI:** 10.1371/journal.pntd.0003051

**Published:** 2014-07-31

**Authors:** Kenji Ishida, Melissa Varrecchia, Giselle M. Knudsen, Emmitt R. Jolly

**Affiliations:** 1 Department of Biology, Case Western Reserve University, Cleveland, Ohio, United States of America; 2 Center for Global Health and Diseases, Case Western Reserve University, Cleveland, Ohio, United States of America; 3 Department of Pharmaceutical Chemistry, University of California, San Francisco, San Francisco, California, United States of America; McGill University, Canada

## Abstract

Schistosomiasis is a chronically debilitating disease caused by parasitic worms of the genus *Schistosoma*, and it is a global problem affecting over 240 million people. Little is known about the regulatory proteins and mechanisms that control schistosome host invasion, gene expression, and development. Schistosome larvae, cercariae, are transiently free-swimming organisms and infectious to man. Cercariae penetrate human host skin directly using proteases that degrade skin connective tissue. These proteases are secreted from anucleate acetabular glands that contain many proteins, including heat shock proteins. Heat shock transcription factors are strongly conserved activators that play crucial roles in the maintenance of cell homeostasis by transcriptionally regulating heat shock protein expression. In this study, we clone and characterize the schistosome Heat shock factor 1 gene (*SmHSF1*). We verify its ability to activate transcription using a modified yeast one-hybrid system, and we show that it can bind to the heat shock binding element (HSE) consensus DNA sequence. Our quantitative RT-PCR analysis shows that *SmHSF1* is expressed throughout several life-cycle stages from sporocyst to adult worm. Interestingly, using immunohistochemistry, a polyclonal antibody raised against an Hsf1-peptide demonstrates a novel localization for this conserved, stress-modulating activator. Our analysis suggests that schistosome Heat shock factor 1 may be localized to the acetabular glands of infective cercariae.

## Author Summary

Schistosome parasites are the cause of human schistosomiasis and infect more than 240 million people worldwide. Schistosome larvae, termed cercariae, are a free-swimming mobile developmental stage responsible for host infection. These larvae produce enzymes that degrade human skin, allowing them to pass into the human host. After invasion, they continue to evade the immune system and develop into adult worms. The transition from free-swimming larvae in freshwater to invasion into a warm-blooded saline environment requires that the parasite regulate genes to adapt to these changes. Heat shock factor 1 is a well-characterized activator of stress and heat response that functions in cellular nuclei. Using immunohistochemistry, we observed non-nuclear localization for anti-Heat shock factor 1 signal in the secretory glands necessary for the invasive function of schistosome larvae. This observation expands the potential mechanistic roles for Heat shock factor 1 and may aid in our understanding of schistosome host invasion and early development.

## Introduction

Schistosomiasis affects more than 240 million people worldwide, ranking second after malaria in the World Health Organization listing of neglected tropical diseases. [1]–[6]. Resistance to praziquantel, the primary therapeutic used for decades to treat schistosome infection, has been reported [7], and partial efficacy is observed in some patients [8]. Thus, the development of novel drug strategies and alternative treatment options are pressing issues in schistosome research [9].

Understanding host-parasite interactions can lead to novel therapeutic strategies that can interfere with infection or eliminate established infections. For example, topical application of inhibitors against the proteases of infective cercariae can block skin invasion [10], [11]. We have been exploring heat-shock pathway components as a potential essential pathway in larval schistosomes. In protozoan parasites, heat shock proteins are essential for mediating changes in morphology during stage differentiation that are often concurrent with stress-related transitions from insect to mammalian host, or extracellular to intracellular conditions [12]–[17]. In human cancers, the chaperone function of heat shock proteins mediates oncogenic transformation and blocks apoptosis [18], [19]. In our *Schistosoma mansoni* system, heat shock proteins 70 and 89 were identified as abundant components of cercarial secretions used for host invasion [20], [21], suggesting a potential role in host-parasite interactions as well.

We have focused on characterizing Heat shock factor 1 in *S. mansoni* as a route to better understanding the role of heat shock pathway activation in infective larvae. Schistosome infections occur when the skin of a potential mammalian host is exposed to schistosome larvae in freshwater. The larvae penetrate the skin and begin development into adult worms. Identification of suitable targets via drug screening approaches are ongoing, but challenges remain to translate the results of these screens into useful anti-schistosomal drugs [22]–[26]. Therefore, a more thorough understanding of basic schistosome biology and schistosome infection strategies is necessary.

Schistosomes require both molluscan and mammalian hosts for parasite development. The free-swimming and infectious schistosome larvae, cercariae, penetrate human skin with the aid of proteolytic enzymes [21], [27]–[29]. During penetration, cercariae lose their tails, while the cercarial head continues to develop, transforming into the next developmental stage, the schistosomula. After invasion, schistosomula immediately begin host immune system evasion strategies, elongate, and develop into male and female adults. Worms pair in the liver, then travel to the veins of the bladder or small intestine, where they produce eggs, the pathologic agent in schistosomiasis. Once the eggs are excreted and reach fresh water, the eggs hatch into transient and free-swimming miracidia, which invade a molluscan host, develop into mother and daughter sporocysts, and mass produce infectious cercariae [27], completing the life-cycle.

The transformation from infectious cercariae to schistosomula involves not only a morphological change, but also includes a change in temperature (from the temperature of the external water to that of the host body (37°C)) and osmolarity (from relatively hypotonic freshwater to a saline environment in the bloodstream of the human host). Before transforming into schistosomula, cercariae must first breach the host skin barrier. Cercariae penetrate human skin by releasing the contents of two sets of acetabular glands (preacetabular and postacetabular). These glands produce many substances, including the proteolytic enzyme cercarial elastase (which breaks down host skin) and mucins (which enable adhesion to the host skin) [11], [19], [29]–[33]. In addition, these glands contain a conglomerate of other proteins such as the heat shock proteins (HSPs) Heat shock protein 70 (Hsp70), Heat shock protein 90 (Hsp90), and Heat shock protein 60 (Hsp60) [20], [21], [28]. After the acetabular glands release their contents, they atrophy to make space for gut and other organ development [34]. Since schistosomula effectively survive the transformation from cercariae, we reasoned that a heat shock response system could be involved in schistosome invasion, as well as adaptation to and survival in a warm-blooded human host.

The heat shock response pathway is a highly conserved and adaptive response system that has evolved to reduce stress-induced cellular damage (for review, see [35]–[37]). When cells are stressed by elevated temperature or by other cellular insults, HSPs such as Hsp70 bind unfolded proteins to prevent protein aggregation [38] and to maintain cellular integrity and organismal viability. A major regulator of the heat shock pathway is Heat shock factor 1 (Hsf1), a transcriptional activator that is critical for positive regulation of HSP transcript levels such as those of *HSP70* [37], [39]. Under non-stress conditions, HSPs are thought to interact with Hsf1 and sequester its transcriptional activity [40], [41]. Under heat stress conditions, HSPs release Hsf1, allowing it to activate the transcription of *HSP70* and other genes encoding HSPs.

Recently, the view that heat shock factors (HSFs) function solely to regulate the heat shock pathway has been changing [42]. Mounting evidence suggests that HSFs have complex roles in development. In *Drosophila*, the *HSF* gene functions in oogenesis and larval development [43], and in mice, mutations in the *HSF* gene result in developmental defects, infertility, retarded growth and lethality [44]. Similarly, *HSF* promotes an extended lifespan in *Caenorhabditis elegans* [45], [46], and it is also involved in the regulation of apoptosis in cancer cell lines [47], [48].

Parasitic schistosomes have a gene encoding Hsf1 [49], [50] that responds to heat stress [51], [52]. A role for Hsf1 in the transformation from free-swimming cercariae to skin schistosomula has not been described. We reasoned that this transformation involves a heat shock response. To begin to address this question, we verified the existence of an *HSF1* homolog in the infective stage of schistosomes, tested its ability to activate transcription, assessed its expression profile across different schistosome developmental stages, and examined its capacity to bind DNA. We report here the identification of an antibody, raised against a sequence from*Sm*Hsf1, which specifically labels the acetabular glands of invasive cercariae, an observation which may have potential implications for novel functions of *Sm*Hsf1 in schistosome host invasion and development.

## Materials and Methods

### Animals and parasites

Infected *Biomphalaria glabrata* snails (strain NMRI, NR-21962) were obtained from the Biomedical Research Institute (BRI, Rockville, MD). *Schistosoma mansoni* cercariae were shed from *B. glabrata* snails as previously described [53]. Sporocyst stage parasites were dissected from these snails.

### Preparation of schistosomal RNA

Sporocyst, cercaria, and 4-hour schistosomulum, *S. mansoni* were each suspended in TRIzol reagent (Invitrogen, Carlsbad, CA) and homogenized by Dounce homogenization. As per manufacturer's instructions for the PureLink RNA Mini kit (Invitrogen), the samples were then centrifuged, and a phenol-chloroform extraction was performed on the supernatant, followed by DNAse I treatment. The eluted RNA was quantitated on a ND-8000 spectrophotometer (Thermo Scientific, Waltham MA). Adult stage and uninfected *B. glabrata* snail RNA was obtained from the BRI.

### Cloning

Reverse transcriptase polymerase chain reaction (RT-PCR) was performed using mixed schistosome RNA (from sporocyst, cercaria, schistosomulum, and adult stages) and Superscript III/Platinum Taq RT-PCR kit (Invitrogen) with forward primer oKI001 (5′- CATATGATGTATGGTTTCACATCTGGACCTCCTGTA-3′) and reverse primer oKI002 (5′- GAATTCTCATTCCAATTCTTCCTCACAAAAATCAGG-3′) (Integrated DNA Technologies, Coralville, IA) for the schistosome gene Smp_068270 (www.genedb.org) and with cycling conditions: single cycle of (45°C for 30 min, 95°C for 2 min) and 25 cycles of (94°C for 30 sec, 50.4°C for 30 sec, 72°C for 2.5 min) with a final extension at 72°C 10 min. The RT-PCR product was subcloned into the SmaI restriction site of the pGBKT7 vector (Clontech) to make plasmid pKI003, and sequenced (Elim Biopharmaceuticals, Hayward, CA) for verification.

### Yeast transformation and modified yeast one-hybrid

*Saccharomyces cerevisiae* yeast strain AH109 was transformed for a modified yeast one-hybrid experiment (with pKI003 in this study) as previously described [54], [55]. Yeast cells were plated on synthetic dextrose medium lacking tryptophan (SD-Trp). Transformed colonies were patched onto SD-Trp plates overlaid with 1000 µg 5-bromo-4-chloro-3-indolyl-α-D-galactopyranoside (X-α-Gal) and incubated at 30°C for 1 day. The yeast cells were used for a serial dilution growth test, for which the cells were grown to saturation in SD-Trp medium, diluted to a 600 nm absorbance (A_600_) value of 0.85 (“1” in the dilution series), serially diluted from 1 to 10^−5^, and grown on synthetic dextrose medium lacking histidine or adenine (SD-His or SD-Ade) plates at 30°C for 3 and 4 days, respectively.

### Electrophoretic mobility shift assay (EMSA)

Biotin-labeled and non-labeled oligonucleotide probes containing DNA binding sequences were designed and obtained from Integrated DNA Technologies. The double-stranded, biotin-labeled oligonucleotide oKI068(ds)(5′-Biotin-ttagaagccgccgagagatct[aGAAagTTCtaGTAc]agatctacggaagactctcct-3′) contains the genomic DNA sequence 112 base pairs upstream of the translation start site (-112) for Smp_106930, the schistosome *HSP70* homolog; the brackets indicate the heat shock binding element (HSE), which closely resembles the HSE consensus sequence, a repeating inverted pentameric sequence (nGAAnnTTCnGAAn) [42], [44], [56], [57]. Unlabeled oligonucleotides used for competition experiments include: oKI032 (5′-ttagaagccgccgagagatct[aGAAagTTCtaGTAc]agatctacggaagactctcct-3′) and oKI033 (reverse complement of oKI032), which match the sequence of oKI068(ds); oKI030 (5′-ttagaagccgccgagagatct[cGAAtTTCgaCTAg]agatctacggaagactctcct-3′) and oKI031 (reverse complement of oKI030), which contain the genomic sequence 239 base pairs upstream of the translation start site of *SmHSP70* (-239); oKI034 (5′-ttagaagccgccgagagatct[cGAAtTTCg]agatctacggaagactctcct-3′) and oKI035 (reverse complement of oKI034), which contain a shortened binding sequence from -239; oKI036 (5'-ttagaagccgccgagagatct[aCTTagTTCtaGTAc]agatctacggaagactctcct-3') and oKI037 (reverse complement of oKI036), which contain three mutated nucleotides in the first pentameric repeat from -112; double-stranded oligonucleotide oAT012(ds)(5′-gctgaaggat[CTAAAAATAG]gcggatcggc-3′), which contains a DNA binding sequence for the putative schistosome myocyte enhancer factor 2 [54]; and oKI073 (5′-gatcgtcat[aGAAagTTCtaGAAc]gatc-3′) and oKI074 (reverse complement of oKI073), which contain the HSE consensus sequence [57]. To make the oligonucleotide probes double-stranded, matched single-stranded oligonucleotides were incubated at 100°C for 2 minutes, after which the temperature was reduced by 1°C each minute, ending when the temperature reached 30°C. The oligonucleotide names and sequences are summarized in Table S1.

Biotin-labeled DNA was detected using the LightShift chemiluminescent EMSA kit (Thermo Scientific) according to the manufacturer's guidelines. Briefly, 3.5 µg each of purified maltose binding protein (MBP) and MBP-*Sm*Hsf1 fusion protein was incubated with 100 fmol of the biotin-labeled oligonucleotide probes either alone or together with 25 pmol of non-labeled oligonucleotide probes in binding buffer, glycerol, MgCl_2_, poly-dIdC (nonspecific DNA competitor), and NP-40 detergent for 30 minutes. The protein-DNA complexes were run on a 5% native polyacrylamide gel in 0.5×TBE at 200 V for 1 hour, transferred to a nylon membrane in 0.5×TBE at 350 mA for 1 hour, and crosslinked on a CL-1000 Ultraviolet Crosslinker (CVP) at an energy setting of 120 mJ/cm^2^. After crosslinking, the membrane was blocked, incubated with a stabilized streptavidin-horseradish peroxidase conjugate, washed, incubated with luminol/enhancer and stable peroxide solution, and visualized on a CCD camera (Fotodyne, Hartland, WI).

### Comparison of protein sequences

Hsf1 protein sequences from *Schistosoma mansoni* (GeneDB, Smp_068270.2), *Schistosoma japonicum* (GeneDB, Sjp_0064040), *Caenorhabditis elegans* (GenBank: AAS72410.1), *Saccharomyces cerevisiae* (Saccharomyces genome database, strain S288C: YGL073W), *Drosophila melanogaster* (NCBI RefSeq: NP_476575.1), *Xenopus laevis* (NCBI RefSeq: NP_001090266.1), *Mus musculus* (GenBank: AAH94064.1), and *Homo sapiens* (NCBI RefSeq: NP_005517.1), along with the protein sequence corresponding to the conserved domain of Hsf1 (NCBI conserved domains, Cdd:smart00415) [9], [10], [58], were aligned using ClustalW2 (http://www.ebi.ac.uk/Tools/msa/clustalw2/) [7], [8] using the default parameters. TreeViewX software was used to generate a phylogram.

### Quantitative reverse transcription polymerase chain reaction (qRT-PCR)

Primers specific to Smp_068270.2 (forward primer oKI040: 5′-TGGTAATGACGAGTGTGACGTA-3′, reverse primer oKI042: 5′-TCAACATTAAGGCCTACAGGAAA-3′) were designed using the Primer3 web applet [59], [60]. One microgram each of sporocyst, cercaria, 4-hour schistosomulum, and adult stage RNA was subjected to a reverse transcriptase reaction with oligo dT (Promega), and 50 ng of the resulting cDNA was used for a relative ΔΔC_T_ qPCR using SYBR Green PCR Master Mix (Applied Biosystems) and primers oEJ548 (5′-AGTTATGCGGTGTGGGTCAT-3′) and oEJ549 (5′-TGCTCGAGTCAAAGGCCTAC-3′) with cytochrome c oxidase subunit 2 (TC7399, TIGR database) as the reference gene. qRT-PCR products are intron-spanning. All experiments were done in triplicate. A two-tailed t-test was applied to ΔC_T_ values as the statistical test to determine significant differences in transcript expression levels relative to the cercaria stage.

### Recombinant protein purification

Smp_068270.2 was subcloned into pMAL-c5X (NEB) at NdeI and EcoRI by ligation using T4 DNA ligase (NEB, Ipswitch, MA) to make plasmid pKI058, and transformed into BL21(DE3) chemically competent *E. coli* bacterial cells (Invitrogen). BL21(DE3) cells carrying either plasmid pKI058 or empty vector pMAL-c5X were induced with isopropyl β-D-1-thiogalactopyranoside (IPTG, Sigma-Aldrich, Saint Louis, MO) to a concentration of 0.4 mM at 37°C for 6 hours, and cell pellets were frozen overnight. Cells were lysed by pulse sonication (Sonifier 250, Branson, Danbury, CT) in a phosphate lysis buffer (50 mM potassium phosphate at pH 8.0, 200 mM NaCl) containing 10 mM phenylmethylsulfonyl fluoride (PMSF) and 100 µL Halt Protease Inhibitor Cocktail (Thermo Scientific). The lysate was cleared by centrifugation at 10,000×*g* for 30 minutes at 4°C (Sorvall), and the cleared supernatant was incubated with amylose resin beads (NEB) at 4°C overnight with gentle rocking. Purified protein (MBP and MBP-*Sm*Hsf1, respectively, from pMAL-c5X and pKI058) was eluted from the amylose beads (50 mM potassium phosphate at pH 8.0, 200 mM NaCl, 10 mM maltose), dialyzed against 3 changes of protein storage buffer (20 mM HEPES pH 7.9, 100 mM KCl, 0.1 mM EDTA, 1 mM DTT, 50% glycerol) Slide-a-lyzer, Thermo Scientific) and concentrated with 30k and 50k MWCO Amicon columns (Millipore, Billerica, MA). The proteins were quantified using the Bradford reagent (Bio-Rad, Hercules, CA) and an ND-8000 spectrophotometer (Thermo Scientific).

### Custom antibody production

A polyclonal antibody raised in New Zealand white rabbits against the peptide with sequence Cys-KYKKEPIRKQHKI from Smp_068270 (*Sm*Hsf1) was designed and purchased (Pacific Immunology, Ramona, CA). The peptide sequence used for antibody production was aligned against the NCBI schistosome protein database (protein BLAST) to prevent production of an antibody cross-reactive with other schistosome proteins. IgG was purified from the pre-immune serum using Melon Gel IgG Spin Purification Kit (Thermo Scientific).

### Western blotting

To detect *Sm*Hsf1 protein, 1 µg purified MBP, 1 µg and 7 µg MBP-*Sm*Hsf1 fusion, and approximately 5 µg of cercarial protein extract were resolved on a 5% polyacrylamide gel and transferred to nitrocellulose membranes in ice-cold Towbin transfer solution (25 mM tris, 192 mM glycine, 20% methanol) at 400 mA for 2 hours. Following the transfer, the membranes were blocked in 5% milk dissolved in phosphate buffered saline, 0.1% Tween-20 (PBSTw) on an orbital shaker at room temperature for 1 hour. Purified IgG from pre-immune serum or immune serum was added to a concentration of 0.5 µg/mL, and the membranes were gently rocked at 4°C overnight. The membranes were washed in PBSTw on an orbital shaker for 5, 10, and 15 minutes, after which an HRP-linked goat anti-rabbit secondary antibody (GE Healthcare) was added at a dilution of 1∶2500 in 1% milk/PBSTw, followed by orbital shaking at room temperature for 1 hour and washing in PBSTw. Amersham ECL Western blotting detection reagent (GE Healthcare) was added (2 mL per nitrocellulose membrane) and incubated at room temperature for 1 minute before the membranes were exposed to autoradiography film.

### Immunohistochemistry

A protocol adapted from Collins *et al.* (2011) was used to prepare samples for immunohistochemistry [61]. Briefly, cercariae were fixed for 20 minutes at room temperature in a 4% paraformaldehyde/PBSTw (PBS/0.1% Tween-20) solution, washed in PBSTw, then dehydrated in a methanol/PBSTw series and stored in 100% methanol at −20°C until use. Prior to use, cercariae were rehydrated, digested for 10 minutes at room temperature in permeabilization solution (1×PBSTw, 0.1% SDS, and proteinase K (1 µg/mL)), and washed in PBSTw (all subsequent washes were carried out with nutation at room temperature). Cercariae were re-fixed for 10 minutes at room temperature in a 4% paraformaldehyde/PBSTw solution, and washed in PBSTw. Samples were incubated with rocking in block solution (PBSTw, 5% horse serum (Jackson ImmunoResearch Laboratories, West Grove, PA), 0.05% Tween-20, and 0.3% Triton X-100) for 2 hrs at RT or overnight at 4°C. Samples were incubated with a polyclonal primary rabbit anti-*Sm*Hsf1 antibody (described above) in block solution at a concentration of 0.6 µg/mL or 2.5 µg/mL, overnight at 4°C and washed >2 hrs at room temperature. Samples were then incubated with an Alexa 647 donkey anti-rabbit antibody (Jackson ImmunoResearch Laboratories) at a concentration of 1∶400 or 1∶800 in block solution, overnight at 4°C. Samples were washed in PBSTw (>2 hrs), at room temperature with the second wash containing DAPI (1 µg/mL). After washing, samples were mounted in Slow Fade Gold (Invitrogen, Grand Island, NY). Pre-immune serum IgG (5 µg/mL), and no primary controls were run in parallel with experimental samples. For the no primary controls, samples were incubated in block solution alone during the primary incubation step.

### Imaging

All samples were mounted in Slow Fade Gold mounting media. Samples were imaged on a Zeiss LSM 510 META confocal microscope (Carl Zeiss, Germany) (Plan-Apochromat 63×/1.2 W objective). The Alexa 647 fluorophore was excited with a 633 nm laser and the DAPI with a 405 nm laser. Images were processed using Zeiss LSM Image Browser (Carl Zeiss) or ImageJ.

## Results

### Schistosome Hsf1 is a transcriptional activator

Hsf1 proteins function as activators of transcription. To test whether the Hsf1 protein from schistosomes (Smp_068270.2) is able to activate transcription, we performed a modified yeast one-hybrid analysis as previously described [54], [55]. Briefly, we made an N-terminal fusion of the DNA binding domain of the yeast Gal4 protein (Gal4DBD) with *Sm*Hsf1 to make the fusion protein Gal4DBD-*Sm*Hsf1. Gal4DBD can bind DNA, but it cannot activate transcription because its transactivation domain has been removed. The Gal4DBD-*Sm*Hsf1 fusion protein was expressed in a yeast strain that is auxotrophic for histidine and adenine (see Materials and Methods). Genes for alpha galactosidase (encoded by *MEL1*), histidine metabolism (encoded by *HIS3*), and adenine metabolism (encoded by *ADE2*), are regulated by promoter elements dependent on Gal4 binding and activation; these genes were used as reporters.

**Figure 1:**
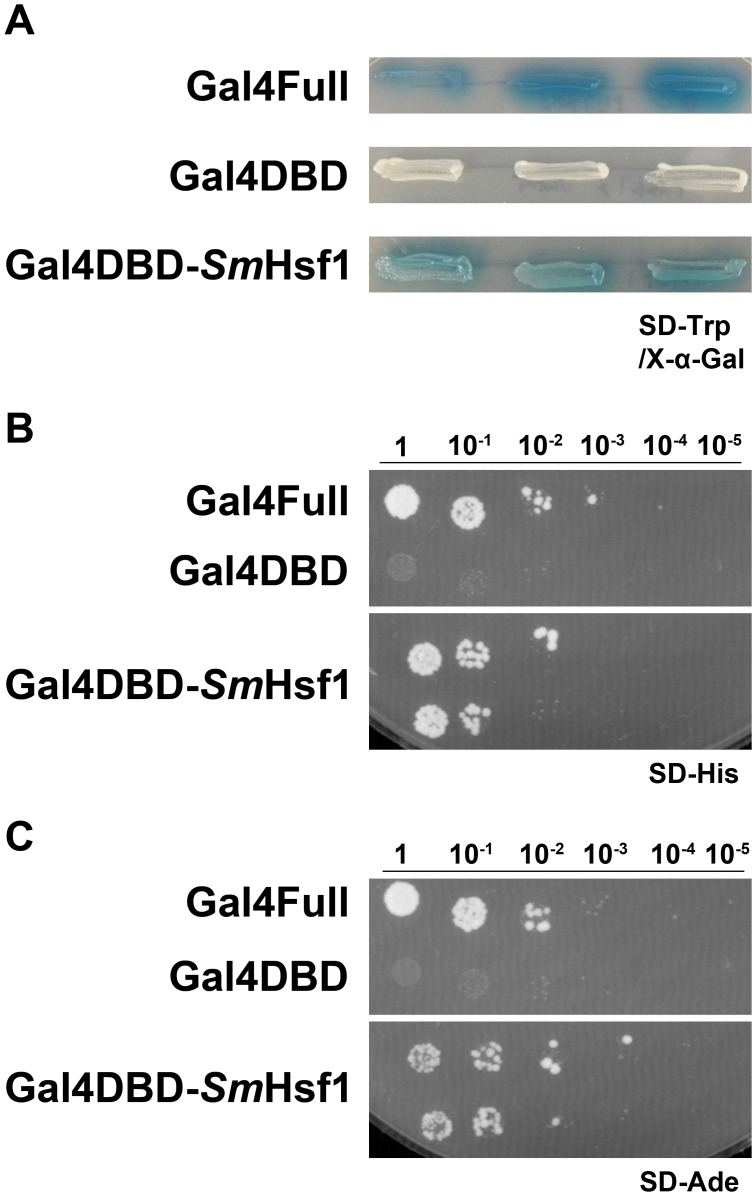
*Sm*Hsf1 can drive transcription in a modified yeast one-hybrid system. Yeast cells expressing *Sm*Hsf1 fused to the Gal4 DNA binding domain (Gal4DBD-*Sm*Hsf1) were patched (A) or serially diluted (B and C, from 1 to 10^−5^) on different selective media to test the ability of *Sm*Hsf1 to activate transcription. The positive control yeast express a complete *GAL4* gene (Gal4Full) and the negative control yeast express the *GAL4* DNA binding domain alone (Gal4DBD). (A) Blue color on SD-Trp with X-α-Gal indicates expression of the *MEL1* reporter gene. (B and C) Growth on the SD-His and SD-Ade plates indicates expression of the *HIS3* and *ADE2* reporter genes, respectively, and are essential for cell viability.

Expression of the Gal4DBD-*Sm*Hsf1 protein in yeast cells resulted in the induction of the *MEL1* reporter gene, which was visualized by blue-colored yeast cells on SD-Trp/X-α-Gal plates (Figure 1A). To test whether *Sm*Hsf1 could also induce *HIS3* and *ADE2* reporter gene expression, yeast cells were selected for growth and viability by a serial dilution assay on synthetic medium lacking the respective nutritional marker (SD-His, SD-Ade). Yeast not expressing the selectable markers cannot survive. We found that yeast cells expressing Gal4DBD-*Sm*Hsf1 protein were viable and conferred histidine and adenine prototrophy to these cells (Figure 1B, 1C). Yeast cells expressing only the Gal4DBD were unable to induce activity from any reporter: they showed no blue color on SD-Trp/X-α-Gal plates and were not viable on SD-His and SD-Ade plates, while the positive control yeast cells expressing the full length Gal4 activator (Gal4Full) showed blue color on SD-Trp/X-α-Gal plates and were viable on SD-His and SD-Ade plates (Figure 1). These data demonstrate that *Sm*Hsf1 functions as a transcriptional activator.

### *Sm*Hsf1 recognizes the heat shock DNA binding element from the schistosome *HSP70* promoter

**Figure 2:**
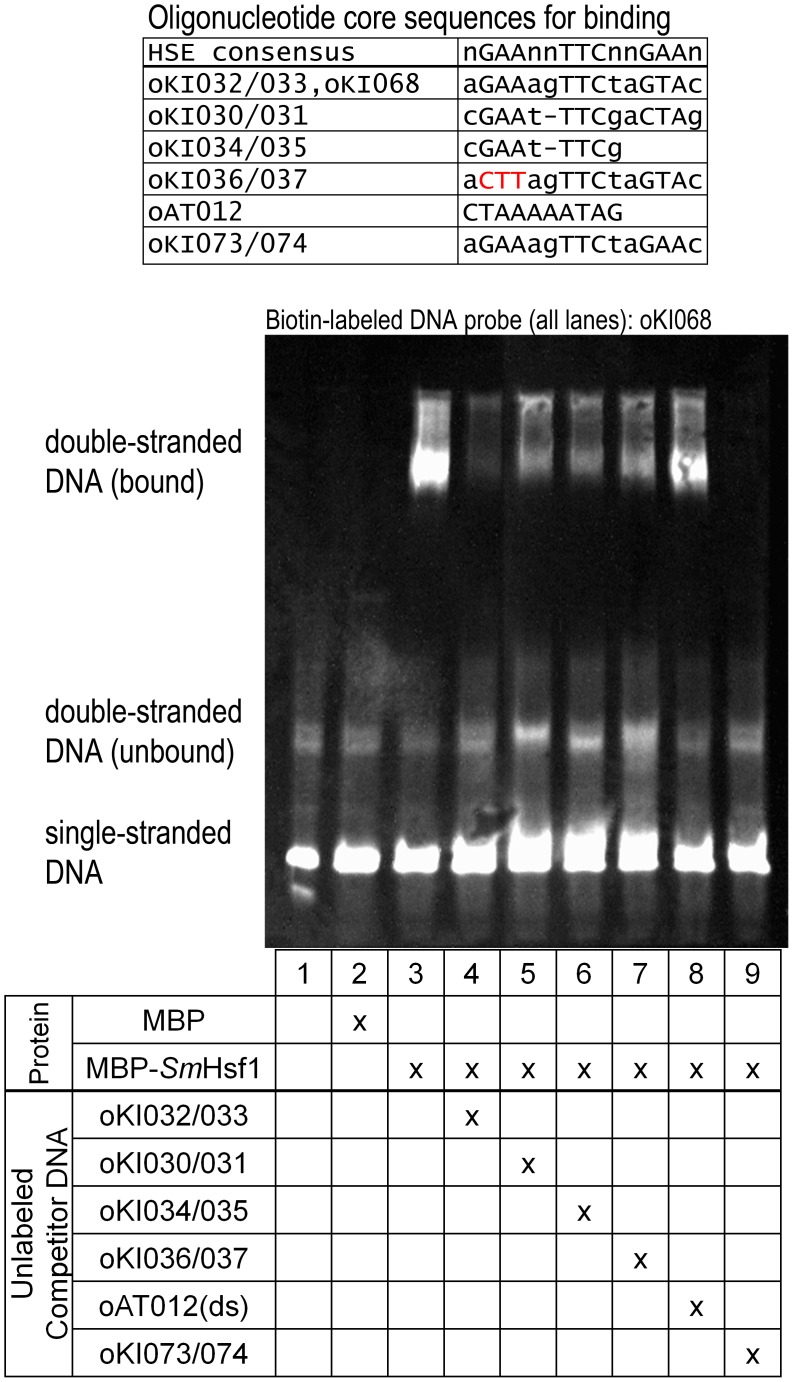
*Sm*Hsf1 binds the heat shock binding element from the schistosome *HSP70* promoter. Recombinant *Sm*Hsf1 cloned as a fusion protein with maltose binding protein (MBP-*Sm*Hsf1), or maltose binding protein (MBP) alone, was incubated with the double-stranded biotin-labeled oligonucleotide oKI068(ds) containing the heat shock binding element sequence from the schistosome *HSP70* promoter. Unlabeled competitor oligonucleotide probes were added at a 250-fold molar excess relative to oKI068(ds) in lanes 4–9. Labeled DNA was detected by chemiluminescence. Oligonucleotide sequences for the probes are shown in Table S1.

**Figure 3:**
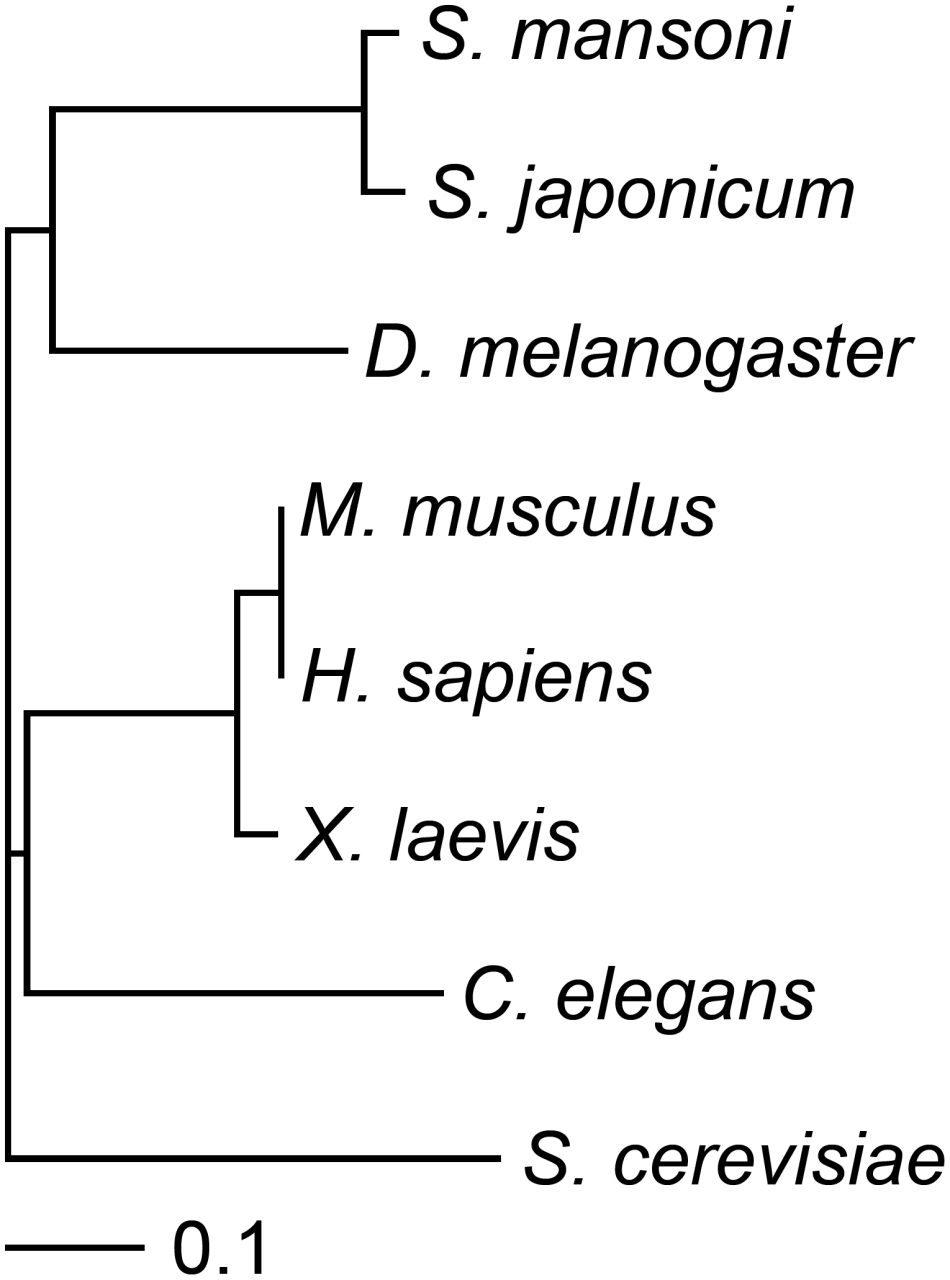
Phylogram of *Sm*Hsf1. Protein sequences of the Hsf1 conserved domain from various species were aligned using ClustalW2, and the phylogram was generated using TreeViewX software. The following settings were used for the protein alignment: Protein Weight Matrix (Gonnet); Gap open (10); Gap extension (0.20), Gap distances (5); No end gaps allowed; Single iteration; Clustering (NJ).

Heat shock factors recognize promoter sequences that regulate several heat shock response genes (such as *HSP70*) by binding to heat shock factor DNA binding elements (HSEs) that consist of repeating inverted pentameric sequences: nGAAnnTTCnnGAAn [42], [56]. We tested whether *Sm*Hsf1 can bind to the HSE located 112 base pairs from the translation start site of the *SmHSP70* gene using an electrophoretic mobility shift assay (EMSA) (Figure 2). Recombinant, purified maltose binding protein-schistosome Hsf1 (MBP-*Sm*Hsf1) fusion protein was incubated with a double-stranded DNA (dsDNA) oligonucleotide probe, oKI068(ds), containing the HSE from the *SmHSP70* promoter (Figure 2, lane 3). The dsDNA oligonucleotide sequence was labeled with biotin for chemiluminescent EMSA detection (see Materials and Methods). Unlabeled dsDNA oligonucleotide probes were used for competition experiments and matched the following: 1] an HSE sequence found at -239 base pairs from the *SmHSP70* translation start site (DNA oligonucleotide pair oKI030/031), 2] an HSE sequence found at -112 base pairs from the *SmHSP70* translation start site (DNA oligonucleotide pairs oKI032/033), 3] an HSE sequence found at -239 base pairs from the *SmHSP70* translation start site lacking the third pentameric repeat (DNA oligonucleotide pair oKI034/035), 4] an HSE sequence found at -112 base pairs from the *SmHSP70* translation start site with three base pairs of the first pentameric repeat mutated (DNA oligonucleotide pair oKI036/037), 5] a negative control sequence reported to bind the putative schistosome Myocyte enhancer factor 2 (dsDNA oligonucleotide oAT012(ds)) [54]; and the HSE consensus sequence (DNA oligonucleotide pair oKI073/074) (Figure 2, lanes 4-9) [57]. Consistent with previous findings [49], [57], we found that MBP-*Sm*Hsf1 binds the HSE from the schistosome *HSP70* promoter. Our competition experiments show that MBP-*Sm*Hsf1 also recognizes the consensus HSE with great affinity (Figure 2, lane 9). The HSE consensus sequence is found in the promoter region of *Drosophila HSP70* [6], [62]. This led us to compare the protein sequence of the conserved domain (which contains the DNA binding domain) of *Sm*Hsf1 to that of Hsf1 from other species using ClustalW2 (Figure 3). Our analysis showed the expected result that Hsf1 from *Schistosoma mansoni* and the related species, *Schistosoma japonicum*, cluster together. However, these flatworm Hsf1 sequences appear to cluster more closely to sequences from *Drosophila* than to those from other organisms, including the roundworm *C. elegans*.

### *SmHSF1* is expressed across schistosome developmental stages

**Figure 4:**
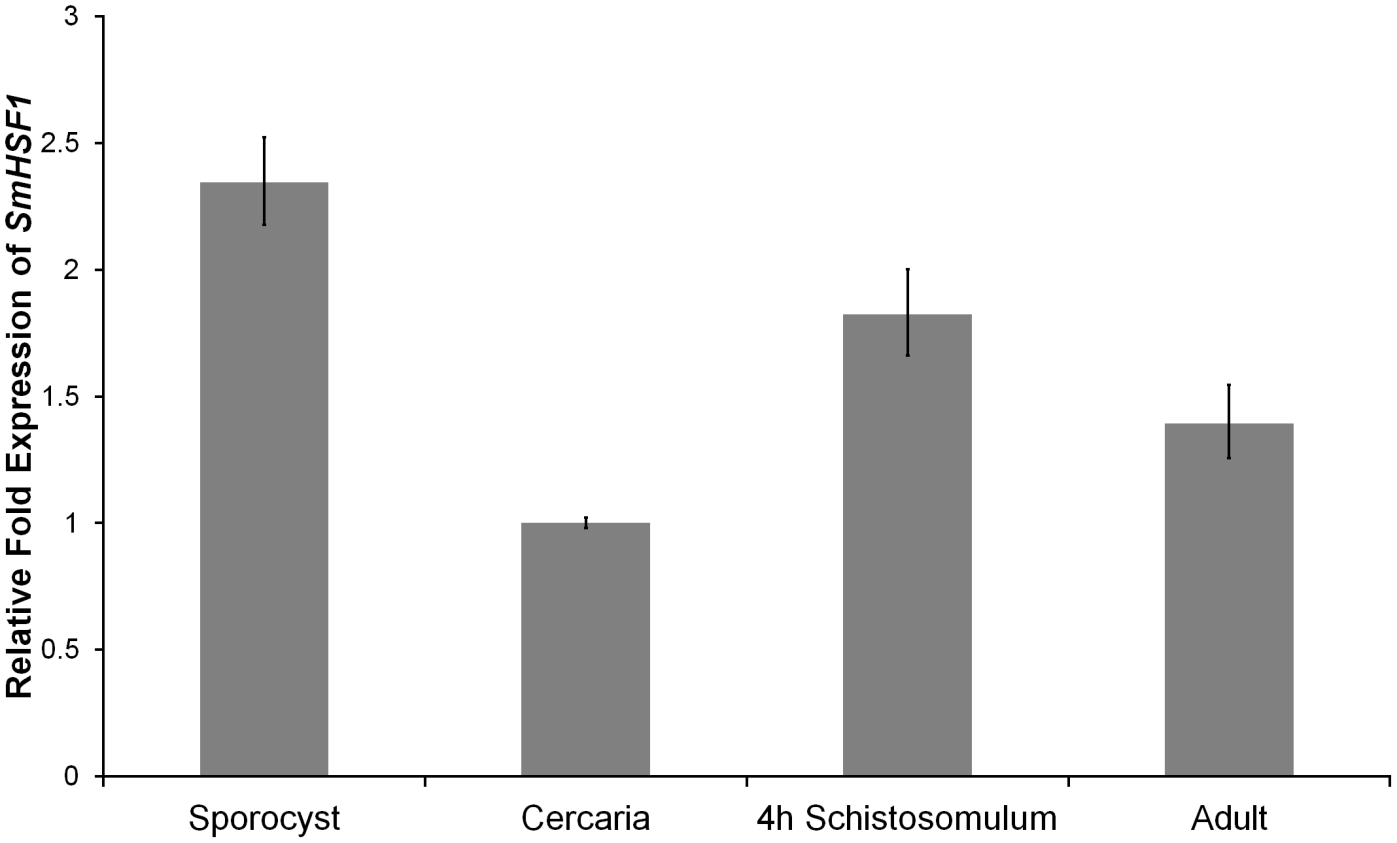
*SmHSF1* is expressed across several schistosome life-cycle stages. qRT-PCR of *SmHSF1* transcript was performed on sporocyst, cercaria, 4-hour schistosomula, and adult stages with 3 replicates. Cytochrome c oxidase was used as the reference gene and cercaria as the reference stage. The ΔΔC_T_ method was used to analyze the qRT-PCR data.

To determine the expression level of *SmHSF1* during schistosome development, we used quantitative reverse transcription PCR (qRT-PCR) to assess *SmHSF1* transcript levels from sporocyst, cercaria, 4-hour schistosomula, and adult stages. Relative to the cercaria stage, *SmHSF1* was expressed 2.3, 1.8, and 1.4-fold in sporocyst, 4-hour schistosomula, and adult stages, respectively (Figure 4, p<0.05). Thus, for all schistosome developmental stages analyzed, *SmHSF1* is expressed. Values of ΔC_T_ were used for a two-tailed t-test to determine significant differences in expression levels.

### A polyclonal antibody detects the *Sm*Hsf1 protein

**Figure 5:**
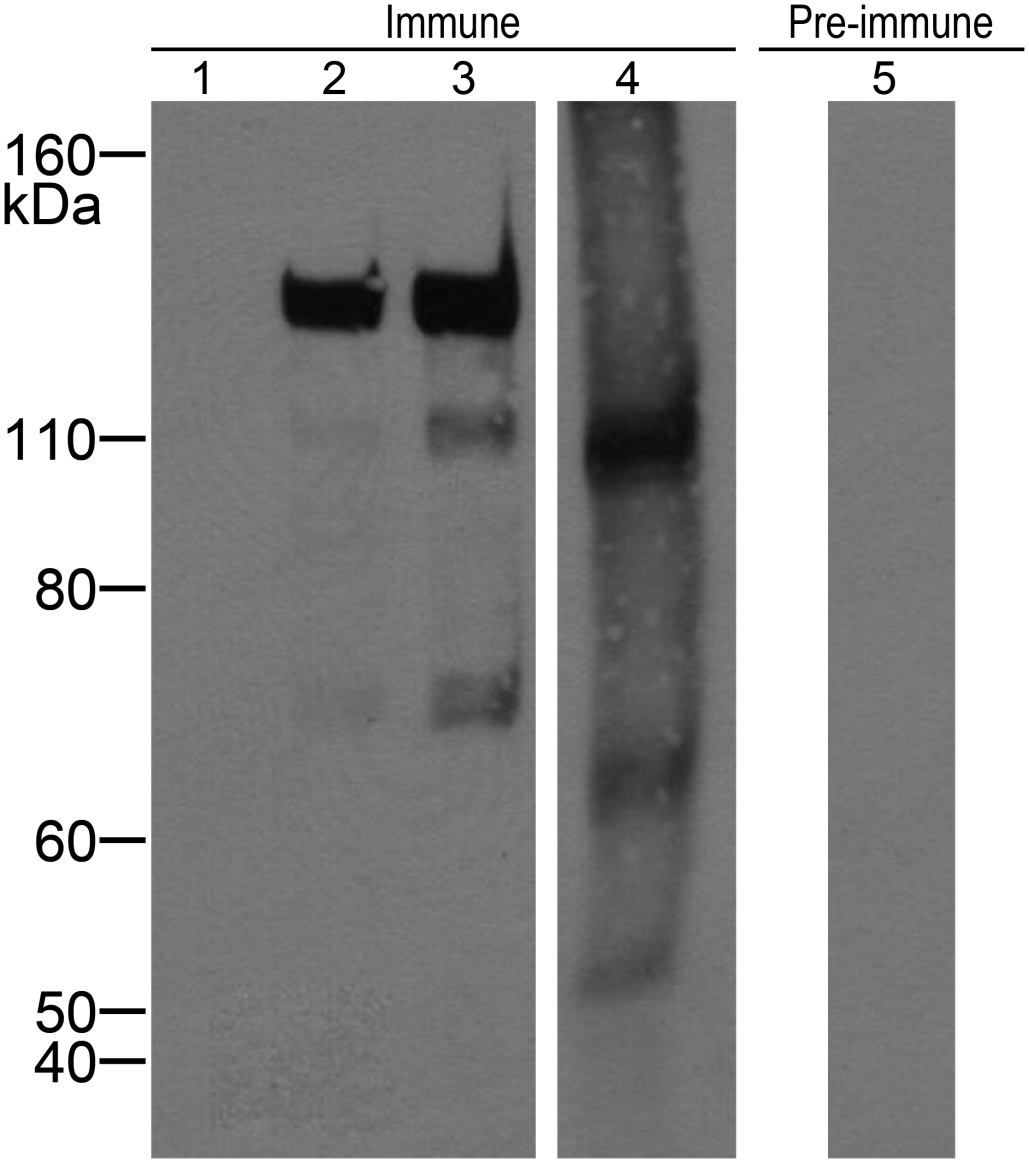
The *Sm*Hsf1 antibody recognizes the *Sm*Hsf1 protein. Purified IgG from *Sm*Hsf1-immunized rabbit bleeds (lanes 1–4) or pre-immune serum (lane 5) were used in a Western blot to test for reactivity against bacterially expressed recombinant proteins and cercarial extract (lane 1), 1 µg MBP negative control (lane 2), 1 µg MBP- *Sm*Hsf1 fusion protein (lane 3), 7 µg MBP- *Sm*Hsf1 fusion protein (lanes 4 & 5), 7 µg cercarial extract.

A custom polyclonal antibody was designed against a peptide sequence (Cys-KYKKEPIRKQHKI) that is common to the known splice variants of the *Sm*Hsf1 protein. Prior to antibody production, the peptide sequence was compared to known schistosome protein sequences by protein BLAST (NCBI) and no statistically significant alignment to other proteins was identified. To test whether the antibody recognizes *Sm*Hsf1, we expressed and purified *Sm*Hsf1 as a fusion protein to the maltose binding protein (MBP-*Sm*Hsf1) to increase solubility and to aid in purification. We performed a Western blot on the recombinant MBP-*Sm*Hsf1 and cercarial protein extract separated by SDS-PAGE, using both pre-immune serum and the purified polyclonal anti-*Sm*Hsf1 antibody (Figure 5). The antibody detected bands at approximately 130, 110, and 70 kDa for the recombinant MBP-*Sm*Hsf1 fusion protein (Figure 5, lanes 2 and 3), all of which were sequence confirmed to contain Hsf1 protein by LC-MS/MS. In cercarial extract, bands were observed at approximately 110, 65, and 50 kDa for the cercarial protein extract, (Figure 5, lane 4), and no non-specific reactivity was observed to the extract using IgG from pre-immune serum (Figure 5, lane 5). In the context of highly abundant background proteins in a complex lysate, it was not possible to detect Hsf1 peptides in the 110, 65, and 50 kDa bands from cercarial extract.

The expected molecular weight of *Sm*Hsf1 is approximately 73 kDa in molecular weight, which when fused to 42 kDa MBP should result in an approximately 115 kDa MBP-*Sm*Hsf1 fusion protein. Since Hsf1 can be highly post-translationally modified [63], we assessed whether treatment with alkaline phosphatase or deglycosidase could collapse the higher molecular weight bands at 130 and 110 kDa, but we observed no band shift. Analysis of MBP signal in these recombinant protein bands by Western blot (Figure S1) demonstrated a similar band pattern to the anti*-Sm*Hsf1 blot. Treatment of recombinant MBP-*Sm*Hsf1 with Factor Xa protease produced the desired cleavage result by liberating the 42 kDa MBP; however, after cleavage, *Sm*Hsf1 is not detected using the antibody against schistosome Hsf1, suggesting the *Sm*Hsf1 protein is not very stable (data not shown).

### Antibody raised against *Sm*Hsf1 localizes to the acetabular glands of *S. mansoni* cercariae

**Figure 6:**
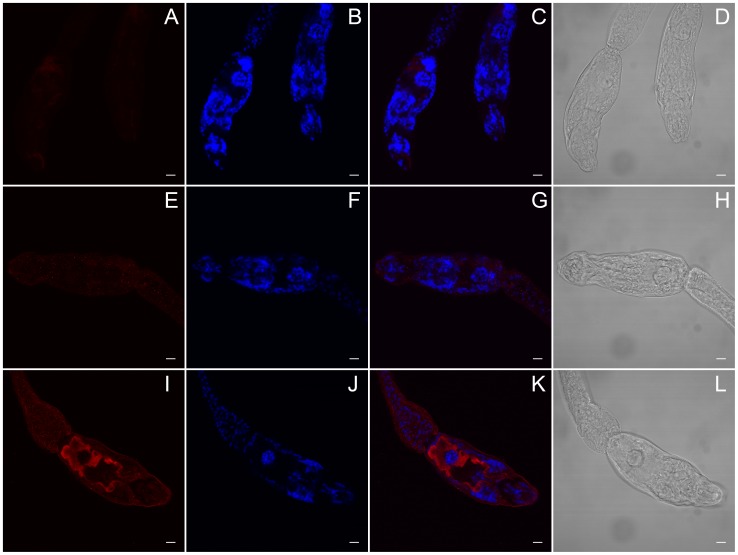
*Sm*Hsf1 may be localized to the acetabular glands in *S. mansoni* cercariae. (A–L) Single, representative confocal sections of cercariae. A custom, rabbit polyclonal primary antibody against *S. mansoni* Heat shock factor 1 protein (*Sm*Hsf1) and a donkey anti-rabbit Alexa 647 secondary antibody were used to detect *Sm*Hsf1 in cercariae. (A–D) No primary negative control. The anterior region (mouth) is located near the bottom of the panels. (E–H) Pre-immune serum IgG negative control. The anterior region is located to the left. (I–L) Anti-*Sm*Hsf1. In panel I, *Sm*Hsf1 is localized to the acetabular glands (red) which traverse the entire head of the cercariae from the posterior (left) to anterior (bottom right). Panels B, F, and J are stained with DAPI. Panels C, G, and K are merged Alexa 647 and DAPI images. Panels D, H, and L are Differential Interference Contrast (DIC) images for each treatment. Scale bar, 10 µm.

**Figure 7:**
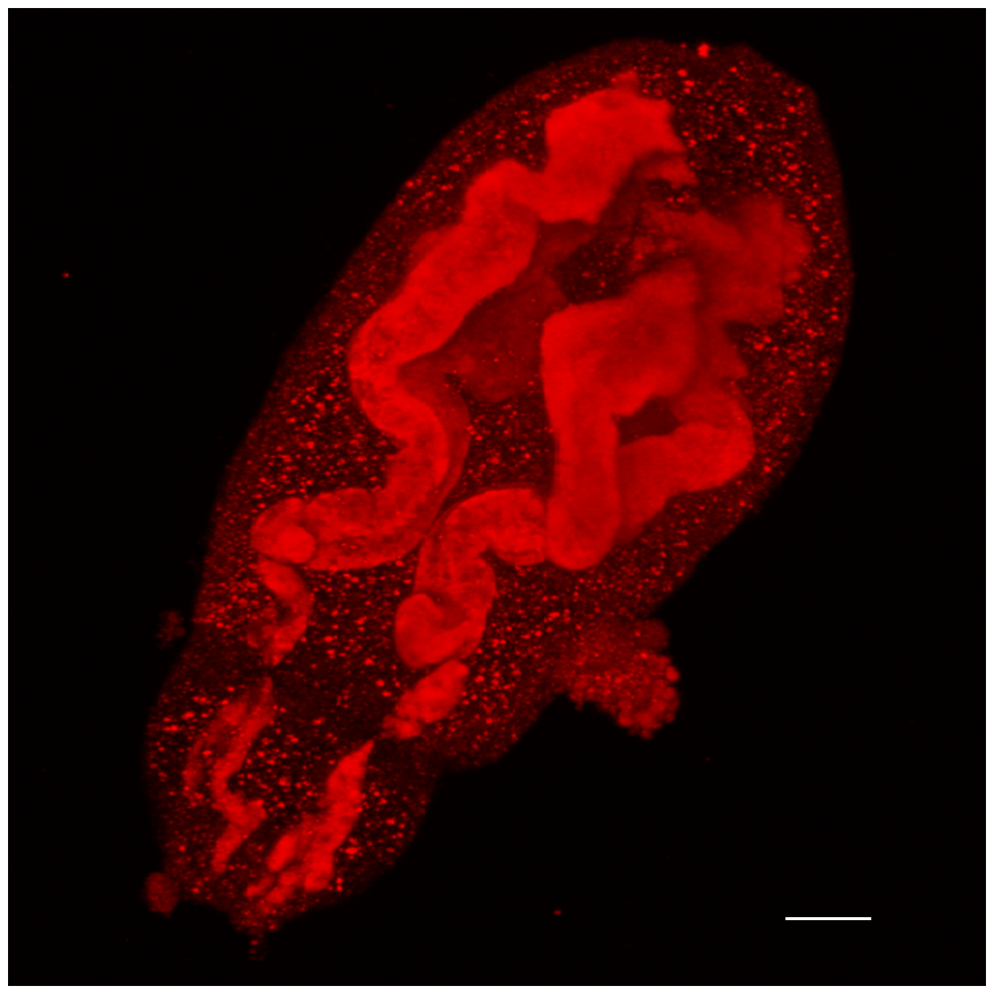
Antibody raised against *Sm*Hsf1 localizes to the acetabular glands extending the entire length of the *S. mansoni* cercarial head. Immune staining, as in Figure 6, was used to localize anti-*Sm*Hsf1 signal (red) to acetabular glands of the *S. mansoni* cercariae. The anterior (mouth) is to the bottom left of the image. The image is a maximum confocal projection, and the magnification is with a 63×/1.2 W objective. Scale bar, 10 µm.

We used indirect immunohistochemistry to determine the location of *Sm*Hsf1 expression in fixed cercariae (Figure 6 and 7; Movie S1-S5). We predicted that *Sm*Hsf1 should produce punctate staining to nuclei throughout the cercariae. We reasoned this because as a transcription factor, Hsf1 is usually localized to the nucleus to induce activation of HSPs [37], [39]. To our surprise, we observed targeted *Sm*Hsf1 localization to the cercarial acetabular glands, which run the length of, and comprise a large percentage of, the cercarial head (Figures 6I, 6K and 7; Movie S1 and S4). Cercarial acetabular glands are composed of three pairs of postacetabular and two pairs of preacetabular glands, whose secretions are thought to be involved in host invasion [31], [64], [65]. These anucleated glands are unicellular, have large fundi located anterior and posterior to the acetabulum (*i.e., pre* and *post*), and have ducts composed of long cellular processes that extend anteriorly to the tip of the oral sucker [64], [66]. Both sets of glands are filled with secretory granules whose contents are thought to be involved in attachment to, and subsequent penetration of, the definitive host. The postacetabular glands contain secretory granules of mucigen, and the preacetabular gland granules contain proteinases [29], [31], [65], [67]. Labeling with the *Sm*Hsf1 antibody occurred along the length of the glands, rather than being restricted to the fundus as might be expected of a transcription factor (Figures 6I and 7; Movie S1 and S4). Our data show that *Sm*Hsf1 is primarily localized to the postacetabular glands. Additionally, *Sm*Hsf1 localization and DAPI staining seem to be mutually exclusive (Movie S3). The no primary and pre-immune controls did not show labeling (Figure 6, panels A and E, respectively).

## Discussion

Hsf1 proteins are highly conserved and well-characterized transcriptional activators. Hsf1 in schistosomes has been previously described [49], [57], [68]. We expand the initial observations on *Sm*Hsf1, and we increase our knowledge of this protein. Hsf1 functions as a transcriptional activator of HSPs in other systems. We cloned the *SmHSF1* gene and assessed whether *Sm*Hsf1 functions as an activator in a heterologous yeast reporter system. Our results confirm that *Sm*Hsf1 is a positive regulator of transcription (Figure 1). Using qRT-PCR, we show that *SmHSF1* transcript is expressed in all stages tested, but that the level of *SmHSF1* is relatively high in sporocysts and relatively low in cercariae. Our findings of transcript levels of *SmHSF1* support the idea that a larger pool of heat shock proteins is required to maintain cell homeostasis in sporocysts because of their elevated protein levels for the mass production of cercariae [69]. Alternatively, this may reflect the general lower transcript levels observed in cercariae, or priming of cercarial transcripts by production of some cercarial transcripts in sporocysts.

The transcript levels of the Hsf1 target, *SmHSP70*, do not change in response to salt or temperature increases in cercariae as expected of a stress response gene [51]. It was speculated that the lack of increase in *HSP70* transcript levels in cercariae prior to the loss of the cercarial tail during transformation is due to tail-dependent inhibitory signals that terminate the transcription of *HSP70* [51]. In support of these observations, we found that the HSP activator, *Sm*Hsf1 protein, is primarily localized to the cercarial acetabular glands, which are unicellular and lack nuclei [64], [66]. We also found that the *Sm*Hsf1 staining does co-localize with the DAPI nuclear stain elsewhere in the cercariae. Thus, it appears that in the absence of nuclear localization, cercarial *Sm*Hsf1 would be unable to induce the transcription of new HSPs, including *HSP70*, despite expectations for this transformation to be a high stress condition. Furthermore, localization of anti-*Sm*Hsf1 signal to cercarial acetabular glands is suggestive of an alternative function for this transcription factor. This motivates our further research into the biological significance of this novel immunolocalization.

The schistosome acetabular glands are long, unicellular structures that produce, store, and release a variety of substances such as mucins and elastases/proteinases to assist in adhesion and invasion of human skin [64]. Curiously, *Sm*Hsf1 has not been detected in cercarial secretions [20], [21], suggesting that *Sm*Hsf1 protein may be bound directly or indirectly to the acetabular cell membrane. At the time of host invasion, the acetabular glands are no longer nucleated [64], [66], raising the possibility of an alternative function for *Sm*Hsf1 beyond a transcriptional role in cercariae and newly transformed schistosomula. *Sm*Hsf1 may be required for the production of chaperone proteins during the development of the glands in early embryonic cercariae in the molluscan host. After fragmentation of the gland nucleus, *Sm*Hsf1 could be released into the cytoplasm, where it can interact with membrane-associated proteins (*Sm*Hsf1 has no known transmembrane domains), facilitating *Sm*Hsf1 to remain bound to the glands during the secretion of other gland contents. A non-nuclear Hsf1 is also observed in non-small cell lung cancer line cells, in which Hsf1 associates with and disables the anti-apoptotic membrane-bound Ralbp1 protein [47], [48]. One scenario is that *Sm*Hsf1 functions to block a related anti-apoptotic factor, allowing apoptosis of the glands to occur. Indeed, in 5-day old schistosomula, the acetabular glands are disintegrated [34], and our immunohistochemical analysis using the anti-*Sm*Hsf1 antibody shows no acetabular *Sm*Hsf1 localization in 5-day old schistosomula (data not shown). *Sm*Hsf1 could be involved in the degradation of acetabular glands in schistosomula, allowing space for the development of organs such as the gut. Additional investigation is required to reveal the mechanism by which *Sm*Hsf1 remains in the glands, and whether it contributes to the controlled disintegration of the glands will be of significant interest. In addition, our data suggest that the anti-*Sm*Hsf1 antibody is primarily localized to the postacetabular glands in cercariae, although we do not exclude the preacetabular glands. It is not clear why there appears to be a preference for the postacetabular glands, responsible for the deposition of cercarial mucins and for providing an adhesive substrate for the parasite to remain attached to host skin [31], [64].

Hsf1 and HSPs could play even broader roles in schistosome developmental regulation. Hsp70 was identified as being responsible for mediating the association and dissociation of the 26S proteasome in mouse embryonic fibroblasts [16]. The 26S proteasome is a conserved set of proteins that function in protein turnover and protein recycling, cell cycle progression through degradation of cyclins, and modulation of cell death [12]–[15], [17]. A major component of the 26S proteasome, the 20S proteasome is reported to bind tightly to Hsp90 in schistosomes, and it has different forms of reactive subunits in cercariae relative to newly transformed schistosomula [18], again connecting the heat shock pathway to cellular development. Examination of the role of *Sm*Hsf1 and HSPs during and after the invasion of human skin not only represents the study of a potentially novel developmental regulatory mechanism, but it can also help to identify key proteins necessary for parasite invasion and development that can be used as therapeutic targets to decrease schistosomula viability in the host. Alternatively, inhibiting the heat shock pathway in cercariae may have unpredictable effects on the ability of schistosomes to infect their host. The heat shock system used by schistosome cercariae during host invasion may also apply to other parasites that undergo environmental transitions, such as *Ancylostoma duodenale* (hookworm), which transition from soil to host, or *Toxoplasma gondii*, which transition from extracellular to intracellular during muscle and brain invasion.

Our data demonstrate that the antibody raised against a peptide in *Sm*Hsf1 can recognize *Sm*Hsf1, but we cannot rule out the possibility that it does not interact with another cercarial protein, and that another protein is responsible for this unique localization to the acetabular glands. Given that our data support previous evidence that the *Sm*Hsf1 protein is extremely insoluble and unstable [49], we were unable to statistically identify the protein in cercariae using mass spectrometry. However, if a protein other than *Sm*Hsf1 is responsible for this novel acetabular localization, this observation provides a cellular marker and an excellent impetus to begin to explore molecular factors in acetabular regulatory mechanisms, as well as an opportunity to further explore host-parasite interactions and parasite development. Understanding the mechanisms for schistosome invasion and development can promote the discovery of novel treatments to combat parasitic infections.

## Supporting Information

Figure S1 **MBP antibody recognizes the MBP-** ***Sm*** **Hsf1 fusion protein.** An antibody against MBP (HRP-conjugated; Abcam, ab49923) was used in a Western blot to probe for MBP in recombinant proteins prepared from *E. coli*. (lane 1) 5 µg MBP positive control, (lane 2) 5 µg intact MBP-*Sm*Hsf1 fusion protein, (lane 3) 5 µg recombinant MBP-*Sm*Hsf1 cleaved with Factor Xa. Signal was detected by chemiluminescence (Pierce ECL western blotting substrate, 32209).
(TIF)

Movie S1 **Anti-** ***Sm*** **Hsf1 antibody is localized to the cercarial head.** Z-stack images of an *S. mansoni* cercarial head showing anti-*Sm*Hsf1 localization. The anterior end the cercarial head is toward the bottom right.
(AVI)

Movie S2 **DAPI staining of the cercarial head.** Z-stack images of an *S. mansoni* cercarial head stained with DAPI. The anterior end of the cercarial head is toward the bottom right.
(AVI)

Movie S3 **Anti-** ***Sm*** **Hsf1 and DAPI staining in the cercarial head.** Merged z-stack images of an *S. mansoni* cercarial head showing anti-*Sm*Hsf1 localization and DAPI staining. The anterior end of the cercarial head is toward the bottom right.
(AVI)

Movie S4 **Rotational view of the cercarial head showing anti-** ***Sm*** **Hsf1 localization.** Maximum projection images are shown. The anterior end of the cercarial head is toward the bottom.
(AVI)

Movie S5 **Rotational view of the cercarial head stained with DAPI.** Maximum projection images are shown. The anterior end of the cercarial head is toward the bottom.
(AVI)

Table S1 **Names and sequences of oligonucleotides used for EMSA.**
(XLS)
