# Supplementary figures and images for: Immunolocalization of Anti-Hsf1 to the Acetabular Glands of Infectious Schistosomes Suggests a Non-Transcriptional Function for This Transcriptional Activator
Source: PLoS Negl Trop Dis. 2014 Jul 31;8(7):e3051. doi: 10.1371/journal.pntd.0003051 (PMC4117452; doi:10.1371/journal.pntd.0003051)

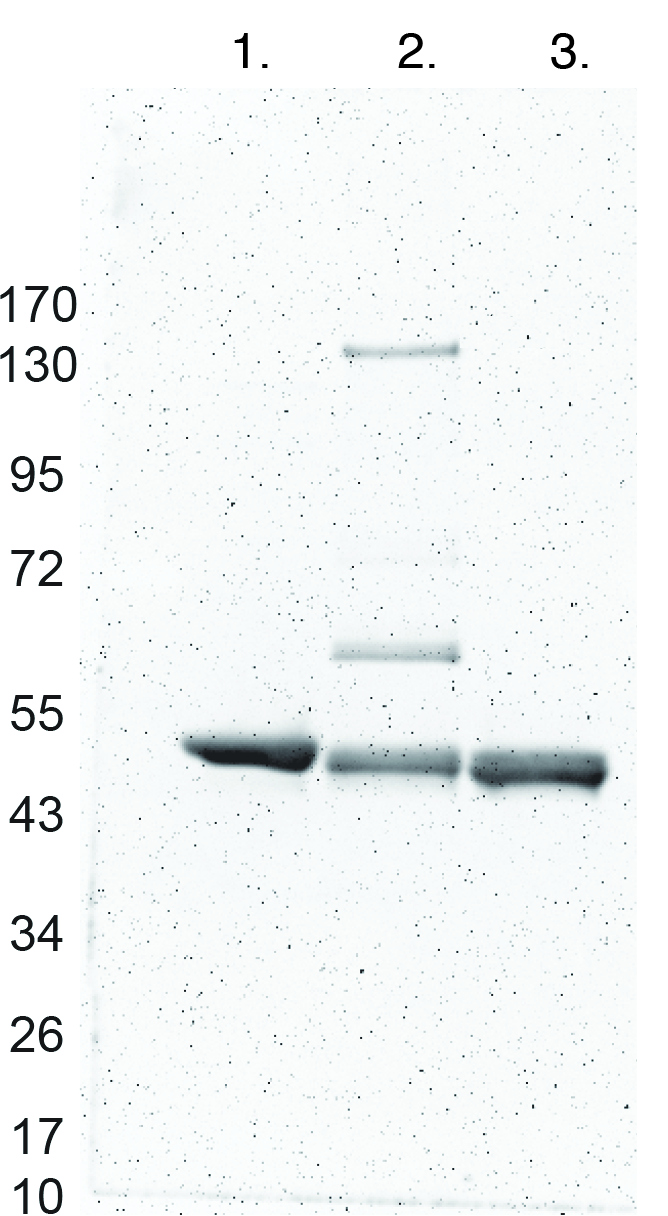

Supplement: Figure S1 — MBP antibody recognizes the MBP- Sm Hsf1 fusion protein. An antibody against MBP (HRP-conjugated; Abcam, ab49923) was used in a Western blot to probe for MBP in recombinant proteins prepared from E. coli. (lane 1) 5 µg MBP positive control, (lane 2) 5 µg intact MBP-SmHsf1 fusion protein, (lane 3) 5 µg recombinant MBP-SmHsf1 cleaved with Factor Xa. Signal was detected by chemiluminescence (Pierce ECL western blotting substrate, 32209). (TIF) [file pntd.0003051.s001.tif]
